# HSP27 Protein Dampens Encephalomyocarditis Virus Replication by Stabilizing Melanoma Differentiation-Associated Gene 5

**DOI:** 10.3389/fmicb.2021.788870

**Published:** 2021-11-26

**Authors:** Xiangrong Li, Ruixian Ma, Bei Wu, Yuhui Niu, Hongshan Li, Dianyu Li, Jingying Xie, Adi Idris, Ruofei Feng

**Affiliations:** ^1^Key Laboratory of Biotechnology and Bioengineering of State Ethnic Affairs Commission, Biomedical Research Center, Northwest Minzu University, Lanzhou, China; ^2^School of Pharmacy and Medical Science, Menzies Health Institute Queensland, Griffith University, Southport, QLD, Australia

**Keywords:** HSP27, encephalomyocarditis virus, MDA5, 2C^pro^, 3A^pro^

## Abstract

Heat shock proteins (HSPs) are a protein family that respond to physiological stress, such as heat, starvation, and infection. As cellular protein chaperones, they play an important role in protein folding, assembly, and degradation. Though it is well known that HSP27 is involved in a range of viral infections, its role during an encephalomyocarditis virus (EMCV) infection is not known. Here, we report that EMCV degrades HSP27 and that EMCV proteins 2C^pro^ and 3A^pro^ are primarily responsible for its degradation. Consequently, loss of cellular HSP27 augmented EMCV proliferation, an effect that could be reversed upon HSP27 overexpression. Importantly, we found that HSP27 positively regulated EMCV-triggered type I interferon (IFN) production. Moreover, overexpression of 2C^pro^ and 3A^pro^ significantly blocked type I IFN production. We also found for the first time that HSP27, as a molecular chaperone, can specifically interact with MDA5 and stabilize the expression of MDA5. Collectively, this study shows that HSP27 dampens EMCV infectivity by positively regulating EMCV-triggered retinoic acid-inducible gene (RIG)-I-like receptor (RLR)/melanoma differentiation-associated gene 5 (MDA5) signal pathway, while EMCV proteins 2C^pro^ and 3A^pro^ interact with HSP27 and degrade HSP27 protein expression to allow EMCV proliferation. Our findings provide further mechanistic evidence for EMCV partaking in immune escape mechanisms, and that 2C^pro^ and 3A^pro^ could serve as potential antiviral targets.

## Introduction

Encephalomyocarditis virus (EMCV) is an important zoonotic pathogen with global distribution, which can cause an acute infectious disease characterized by encephalitis, myocarditis, or pericarditis in artiodactyls, rodents, and even primates (Carocci and Bakkali-Kassimi, 2012). A serological survey found high EMCV antibody-positive rates in Europe, Asia, America, and Africa (Maurice et al., 2005; Feng et al., 2015). Indeed, the global outbreak and prevalence of EMCV have caused serious economic losses to the development of the livestock industry (Feng et al., 2015). EMCV genome contains a full-length 7.8-kb open reading frame (ORF) that encodes a polypeptide consisting of 2,292 amino acids. The polypeptide is then cleaved into a precursor protein L^pro^, four structural proteins (VP1, VP2, VP3, and VP4), and eight non-structural proteins (2A, 2B, 2B*, 2C, 3A, 3B, 3C, and 3D) by virally encoded 2A and 3C proteases (Carocci and Bakkali-Kassimi, 2012). The cytoplasmic retinoic acid-inducible gene (RIG)-I-like receptors (RLRs), especially melanoma differentiation-associated gene 5 (MDA5), play a key role in the innate immune response against EMCV (Loo et al., 2008; Zhu et al., 2018). Like many viruses, EMCV also encodes proteins to subvert and suppress host immune responses by antagonizing the RLR/MDA5 signaling pathway (Li et al., 2021). 3C^pro^ can interfere with the formation of the TANK-TBK1-IKKε-IRF3 complex to inhibit type I interferon (IFN) signaling (Huang et al., 2017) and suppress the pro-inflammatory TRAF6-mediated NF-κB signaling pathway (Huang et al., 2015). 2C^pro^ can interact with MDA5 and inhibit the IFN-β signaling pathway (Li et al., 2019). On the other hand, VP2^pro^ degrades RLR sensors MDA5, MAVS, and their downstream adaptor TBK1’s expression through proteasome and lysosomal pathways (Han et al., 2021).

Heat shock proteins (HSPs) are molecular chaperones that play an important role in protein folding, assembly, and degradation (Tutar and Tutar, 2010; Ikwegbue et al., 2017; Zininga et al., 2018; Bolhassani and Agi, 2019). Many viral proteins have been shown to interact with HSP27 to regulate virus-induced autophagy, type I IFN and NF-κB signaling pathways (Ling et al., 2018; Sun et al., 2018). However, it is not known whether HSP27 plays a role in EMCV infection. We reported that HSP27 was degraded during an EMCV infection *via* EMCV proteins 2C^pro^ and 3A^pro^. We found that HSP27, as a host factor against EMCV infection, positively regulated the production of EMCV-triggered type I IFNs. Moreover, this study shows for the first time that HSP27, as a molecular chaperone, positively regulates EMCV-triggered RLR/MDA5 signal pathway by stabilizing the expression of MDA5. This study reveals a novel role of EMCV 2C^pro^ and 3A^pro^ in antagonizing host antiviral responses, and elucidates the role of EMCV in modulating HSP27 function. Our findings provide further mechanistic evidence for EMCV partaking in immune escape mechanisms, and that 2C^pro^ and 3A^pro^ could serve as potential antiviral targets.

## Materials and Methods

### Cells and Virus

Human non-small cell lung cancer (A549) cells, stable knockdown A549 cell lines of viral 2C^pro^ (shRNA-2C-002), stable knockdown A549 cell lines of viral 3A^pro^ (shRNA-3A-001), and their negative control A549 cell lines (shRNA-NC) by lentivirus infection were cultured at 37°C under 5% CO_2_ in RPMI-1640 (Lanzhou Bailing Biotech) containing 15% new bovine serum (NBS, Lanzhou Minhai Bio-engineering). Baby hamster kidney (BHK-21) cells were cultured at 37°C under 5% CO_2_ in Dulbecco’s modified Eagle’s medium (DMEM, Lanzhou Bailing Biotech) containing 10% new bovine serum (NBS, Lanzhou Minhai Bio-engineering). EMCV strain PV21 (GenBank No: X74312) was propagated and titrated in BHK-21 cells.

### Reagents and Antibodies

NP-40 lysis buffer (P0013F), radioimmunoprecipitation assay (RIPA) lysis buffer (P0013K), protein G Agarose (P2009), antibodies against rabbit IgG (A7016), and GRP78 (AF0171) were purchased from Beyotime (Shanghai, China). Lipofectamine™ 2000 (11668019) was purchased from Thermo Fisher Scientific (Waltham, MA, United States). Polyvinylidene fluoride (PVDF) Membrane (IPVH00010) was purchased from Merck (Darmstadt, Germany). Antibodies against HSP90β (ABP54794) and HSP70 (Abm40042) were purchased from Abbkine (Wuhan, Hubei, China). Antibodies against glyceraldehyde-3-phosphate dehydrogenase (GAPDH) (60004-1-Ig), HA Tag (51064-2-AP), Myc Tag (60003-2-Ig), IRF3 (11312-1-AP), HSP27 (18284-1-AP), MDA5 (21775-1-AP), and MAVS (14341-1-AP) were purchased from Proteintech (Wuhan, Hubei, China). Antibodies against p-IRF3 (29047), TBK1 (38066), and p-TBK1 (5483) were purchased from Cell Signaling Technology (Boston, MA, United States). Anti-VP1 antibody was kindly provided by Dr. Juan Bai (Nanjing Agricultural University, Nanjing, China). Peroxidase affiniPure goat anti-rabbit IgG (H + L) (111-035-003) and anti-mouse IgG (H + L) (115-035-003) antibodies were purchased from Jackson ImmunoResearch Laboratories (West Grove, PA, United States).

### Plasmids, Inhibitors, Small Interfering RNA Oligos, and Transfection

The empty vectors pCMV-HA and pCMV-Myc were provided by the Biomedical Research Center of Northwest Minzu University. The recombinant plasmids Myc-HSP27, Myc-OAS1, Flag-MDA5, HA-VP1, HA-VP2, HA-2A, HA-2C, HA-3A, Myc-VP3, and Myc-3C were all constructed in the lab. The proteasome inhibitor MG132 (S1748) and caspase inhibitor Z-VAD-FMK (C1202) were obtained from Beyotime (Shanghai, China). The autolysosome inhibitor chloroquine (tlrl-chq) and poly (I:C) (tlrl-pic) were obtained from InvivoGen (San Diego, CA, United States). The small interfering RNA (siRNAs) an analog of double-stranded RNA (dsRNA) targeting homo HSP27 were designed and synthesized by Guangzhou Ribo Biotechnology (China). The siRNA sequences are listed in Table 1. A549 cells in 6-well plates were transfected with siRNAs or various plasmids using Lipofectamine™ 2000 according to the manufacturer’s instructions.

**TABLE 1:** Nucleotide sequences of small interfering RNA (siRNA).

Primer names	Target sequences (5′-3′)
si*HSP27*-001	GCTGCAAAATCCGATGAGA
si*HSP27*-002	GGTGCTTCACGCGGAAATA
	

### Virus Infection and Titration of Viral Progeny

For *in vitro* virus infection, the untreated A549 cells or A549 cells transfected with different plasmids were infected with EMCV at a multiplicity of infection (MOI) of 0.01 or 0.1 and cultured at 37°C for 2 h. Then cells were washed with phosphate-buffered saline (PBS) three times, replaced with DMEM containing 3% NBS, and incubated at 37°C for the indicated time. The cells and supernatants were repeatedly frozen and thawed to detect TCID_50_ in BHK-21 cells (using the Reed-Muench method), while the cells collected separately were used for subsequent quantitative real-time polymerase chain reaction (RT-qPCR) or immunoblotting analysis.

### RNA Extraction, Reverse Transcription, and Quantitative Real-Time Polymerase Chain Reaction

According to manufacturers’ protocol, total RNA was extracted from cells using RNAiso Plus (Takara, 9109). Then, 1 μg of total RNA was reverse transcribed using Evo M-MLV RT kit with gDNA clean for qPCR II (Accurate biology, AG11711) to synthesis cDNA. Relative RT-qPCR was performed using the TransStart^®^ Top Green qPCR SuperMix (+Dye II) (Transgen, AQ132-24), and target gene expression was calculated by normalization to GAPDH using the 2^–ΔΔCt^ method. The copy number of the polymerase 3D gene was detected by absolute RT-qPCR using the Premix Ex Taq Probe qPCR (Takara, RR390A). The probe and primer sequences used in this study are described in Table 2.

**TABLE 2:** Nucleotide sequences of the RT-qPCR primers.

Primer names	Primer sequences (5′-3′)
Homo-*HSP27*-qF	CTGACGGTCAAGACCAAGGATG
Homo-*HSP27*-qR	GTGTATTTCCGCGTGAAGCACC
Homo-*GAPDH*-qF	GTCTCCTCTGACTTCAACAGCG
Homo-*GAPDH*-qR	ACCACCCTGTTGCTGTAGCCAA
Homo-*IFN-*α-qF	GCCTCGCCCTTTGCTTTACT
Homo-*IFN-*α-qR	CTGTGGGTCTCAGGGAGATCA′
Homo-*IFN-*β-qF	TTGTTGAGAACCTCCTGGCT
Homo-*IFN-*β-qR	TGACTATGGTCCAGGCACAG
EMCV-probe	(FAM)CACTTCGATCACTATGCTTGCCGTT(Eclipse)
EMCV-*3D*-qF	GTCATACTATCGTCCAGGGACTCTAT
EMCV-*3D*-qR	CATCTGTACTCCACACTCTCGAATG

### Co-immunoprecipitation and Immunoblotting

The transfected cells were lysed in RIPA or NP-40 lysis buffer containing a protease and phosphatase inhibitor cocktail (Beyotime, P1045) for 30 min on ice. And then, the cell lysates were centrifuged at 12,000 × *g* for 30 min at 4°C to remove the debris. For Immunoprecipitation, the cell supernatant was incubated with corresponding antibodies at 4°C overnight, then added Protein G Agarose (Beyotime, P2009) and incubated at 4°C for another 3 h. The bead complexes were washed five times with ice-cold phosphate-buffered saline-tween (PBST) and subjected to immunoblotting, target proteins were fractionated on 10% SDS-PAGE gels and transferred to PVDF membranes (Merck, ISEQ00010) separately, and these membranes were blocked and incubated with the corresponding primary antibodies and peroxidase affinipure goat anti-rabbit or mouse IgG (H + L). Finally, the bound protein bands were detected using Western lightning Plus-ECL kit (PerkinElmer, NEL105001EA) in membranes and visualized with Amersham Imager 600 imaging system (GE).

### Statistical Analysis

Data are displayed as the mean ± standard deviation (SD) of three independent experiments. Statistical significance was determined with one-way analysis of variance (ANOVA) or Student’s *t*-test (*P < 0.05; ^**^P < 0.01; ^***^P < 0.001).

## Results

### Encephalomyocarditis Virus 2C^pro^ and 3A^pro^ Directly Bind and Degrade HSP27

**FIGURE 1:**
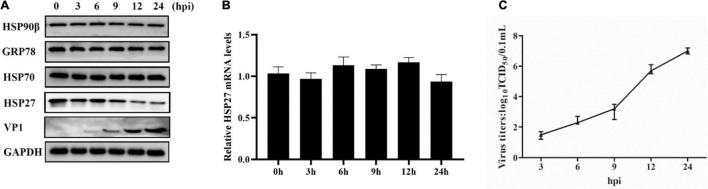
Encephalomyocarditis virus (EMCV) infection degrades HSP27 protein expression in A549 cells. A549 cells were infected with EMCV at an multiplicity of infection (MOI) of 0.1 for 0, 3, 6, 9, 12, or 24 h. **(A)** The expression of HSP27, VP1, HSP90β, GRP78, and HSP70 were analyzed by immunoblotting. GAPDH was used as a loading control. **(B)** *HSP27* mRNA levels were measured by RT-qPCR. **(C)** Cells and supernatant were harvested, and viral titers were determined by TCID_50_ assay (Reed-Muench method).

**FIGURE 2:**
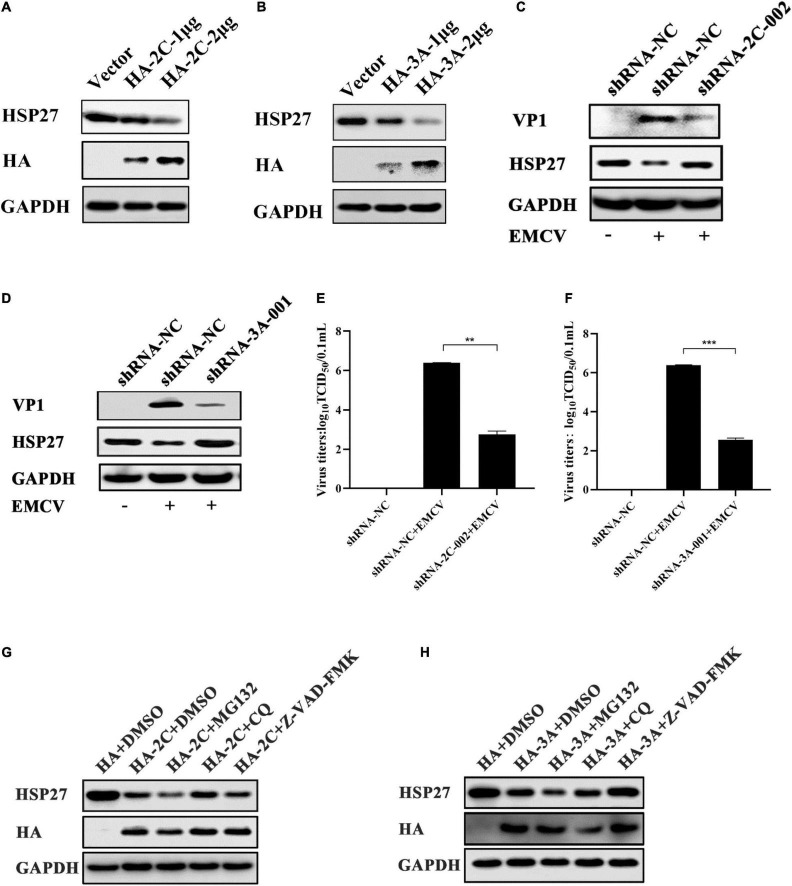
Encephalomyocarditis virus (EMCV) degrades HSP27 protein *via* 2C^pro^ and 3A^pro^. **(A)** A549 cells were transfected with increasing doses of HA-2C (1.0 and 2.0 μg). Immunoblotting was used to analyze HA tag and endogenous HSP27 protein expression. GAPDH was used as a loading control. **(B)** A549 cells were transfected with increasing doses of HA-3A (1.0 and 2.0 μg). Immunoblotting was used to analyze HA tag and endogenous HSP27 protein expression. GAPDH was used as a loading control. The stable knockdown A549 cell lines of viral 2C^pro^ (shRNA-2C-002) or the stable knockdown A549 cell lines of viral 3A^pro^ (shRNA-3A-001) and shRNA-NC cell lines were infected with EMCV at an multiplicity of infection (MOI) of 0.01 for 36 h. **(C,D)** Immunoblotting was used to measure VP1 and HSP27 protein expression, respectively. GAPDH was used as a loading control. **(E,F)** Viral titers were measured by TCID_50_ assay. **(G,H)** A549 cells were transfected with HA-2C or HA-3A (1.0 μg) and then treated with MG132 (10 μM), chloroquine (CQ) (50 μM) or Z-VAD-FMK (20 μM) or DMSO for 6 h, respectively. The expression of HA tag and HSP27 protein were detected by immunoblotting. GAPDH was used as a loading control. Data were represented as mean ± SD of three independent experiments and were measured in technical duplicate, **P < 0.01 and ***P < 0.001.

**FIGURE 3:**
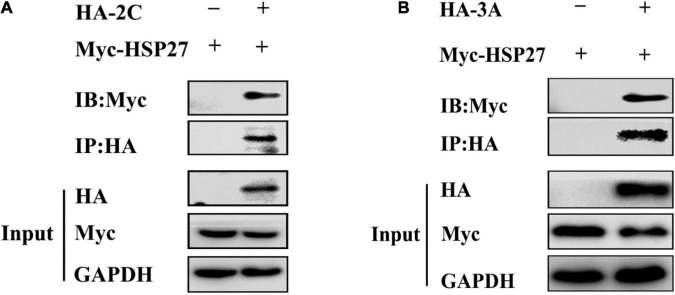
2C^pro^ and 3A^pro^ interact with HSP27. **(A)** A549 cells were co-transfected with Myc-HSP27 together with either pCMV-HA or HA-2C for 36 h before performing co-immunoprecipitation (Co-IP) and immunoblotting with anti-HA antibody. **(B)** A549 cells were co-transfected with Myc-HSP27 together with either pCMV-HA or HA-3A for 36 h before performing Co-IP and immunoblotting with anti-HA antibody.

Lung epithelial A549 cells were infected with EMCV (MOI = 0.1) to explore the expression profile of HSPs in EMCV-infected cells, and the dynamics of HSPs expression were evaluated over a 24 h period. Out of all HSPs tested, HSP27 expression decreased only at the protein level from 9 h post-infection (hpi) onward, suggesting that HSP27 protein expression is lost, presumably *via* degradation during an EMCV infection (Figures 1A–C). We hypothesize that EMCV proteins are responsible for HSP27 degradation. A549 cells were transfected with increasing doses of HA or Myc-labeled EMCV viral proteins, and endogenous HSP27 protein expression was assessed. Only viral proteins 2C^pro^ and 3A^pro^ reduced HSP27 protein levels (Figures 2A,B), whereas VP1^pro^, VP2^pro^, VP3^pro^, 2A^pro^, and 3C^pro^ had no effect (Supplementary Figures 1A–E). We further verified this in stable A549 cell lines engineered to express short hairpin RNA (shRNA) targeted toward viral 2C^pro^ and 3A^pro^. Here, we showed that both shRNA-2C-002 and shRNA-3A-001 significantly reversed the loss of HSP27 expression during EMCV infection (Figures 2C,D). Moreover, when we attenuated the expression of 2C^pro^ and 3A^pro^, respectively, the EMCV VP1 protein and viral titers were significantly reduced (Figures 2C–F), indicating that the expression of 2C^pro^ and 3A^pro^ are significantly positively correlated with EMCV proliferation. To see whether the proteasomal, lysosomal, or caspase-dependent apoptosis pathways play a role in 2C^pro^ and 3A^pro^-mediated HSP27 degradation, we used the proteasome inhibitor MG132, the autolysosome inhibitor chloroquine (CQ), or the pan-caspase inhibitor, Z-VAD-FMK, to evaluate the inhibitory effects of 2C^pro^ and 3A^pro^ on HSP27 protein expression, respectively. Nearly all tested inhibitors failed to reverse the 2C^pro^-mediated reduction of HSP27 (Figure 2G), suggesting that the inhibitory effect of 2C^pro^ on HSP27 was independent of these pathways. However, we found that the reduction of HSP27 protein expression by 3A^pro^ could be reversed upon treatment with Z-VAD-FMK (Figure 2H), indicating that 3A^pro^-mediated HSP27 degradation is dependent on caspase activity. We then wondered whether HSP27 directly interacts with 2C^pro^ and 3A^pro^. Indeed, co-immunoprecipitation (Co-IP) assays confirmed an interaction between HSP27 and 2C^pro^ or 3A^pro^ (Figures 3A,B). Collectively, these results show that 2C^pro^ and 3A^pro^ directly bind and degrade HSP27.

### HSP27 Plays an Antiviral Role Against Encephalomyocarditis Virus

**FIGURE 4:**
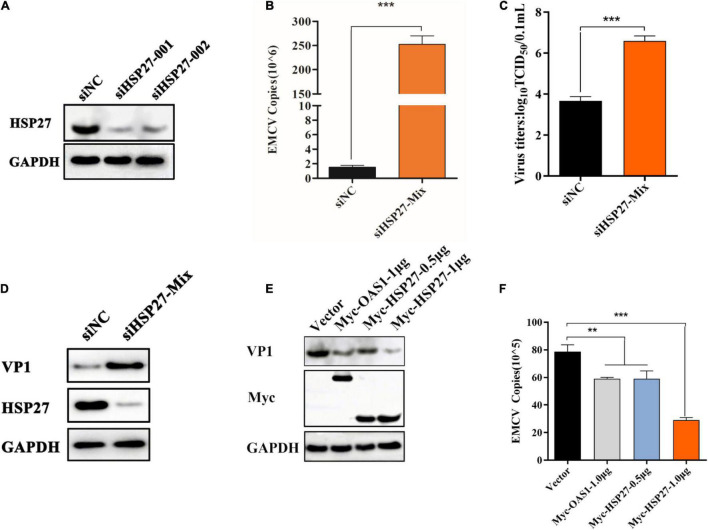
HSP27 has an antiviral role against encephalomyocarditis virus (EMCV). **(A)** A549 cells in 6-well plates cells were transfected with either 150 nmol of siHSP27-001, siHSP27-002, or siNC for 36 h before measuring HSP27 protein by immunoblotting. GAPDH was used as a loading control. A549 cells were transfected with either 150 nmol of siHSP27-Mix or siNC for 36 h before infecting with EMCV at an multiplicity of infection (MOI) of 0.01 for 36 h. **(B)** EMCV copy number was quantified by RT-qPCR, and **(C)** Viral titers were measured by TCID_50_ assay. **(D)** The protein expression of HSP27 and VP1 were analyzed by immunoblotting. GAPDH was used as a loading control. A549 cells were transfected with pCMV-Myc (1 μg), Myc-OAS1 (1 μg), or Myc-HSP27 (0.5/1 μg) plasmids for 24 h before infecting with EMCV at an MOI of 0.01 for 36 h. **(E)** The protein expression of Myc tag and VP1 were analyzed by immunoblotting. GAPDH was used as a loading control. **(F)** Cells and supernatant were harvested, and EMCV copy number was measured by RT-qPCR. Data were represented as mean ± SD of three independent experiments and were measured in technical duplicate, **P < 0.01 and ***P < 0.001.

In light of the observation that EMCV infection and its viral proteins 2C^pro^ and 3A^pro^ could lead to the degradation of HSP27 suggests that HSP27 is involved in dampening EMCV infection. To evaluate this, we designed two siRNAs targeting HSP27 (Figure 4A). Combining both siRNAs enhanced viral growth (Figures 4B,C), corresponding to elevated VP1 expression (Figure 4D). To further confirm the antiviral role of HSP27, A549 cells were transfected with increasing doses of an HSP27 expressing plasmid and then infected with EMCV over 24 h. We also overexpressed 2′,5′-oligoadenylate synthase 1 (OAS1) as a positive control as OAS1 is known to inhibit EMCV proliferation (Kristiansen et al., 2010). Overexpression of either OAS1 or HSP27 reduced VP1 levels in EMCV infected cells (Figure 4E), consistent with lower EMCV copy number and titers (Figure 4F and Supplementary Figure 2), suggesting that HSP27 can dampen EMCV proliferation. Collectively, our data show that HSP27 plays a negative regulatory role in the proliferation of EMCV.

### HSP27 Positively Regulates the Production of Encephalomyocarditis Virus-Triggered Interferon-I

**FIGURE 5:**
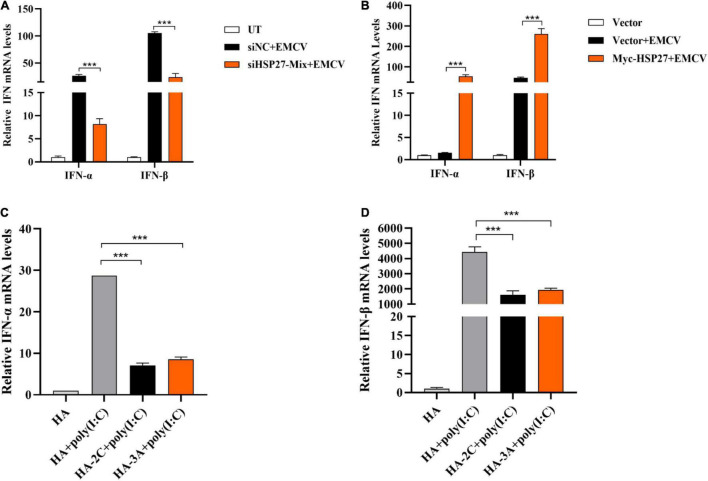
HSP27 positively regulates the production of encephalomyocarditis virus (EMCV)-triggered IFN-I. **(A)** A549 cells were transfected with either 150 nmol of siHSP27-Mix or siNC for 36 h before infecting with EMCV at an multiplicity of infection (MOI) of 0.01 for 36 h. *IFN-*α and *IFN-*β mRNA levels were measured by RT-qPCR. Untreated group (UT) cells were used as control. **(B)** A549 cells were transfected with Myc-HSP27 (1 μg) or pCMV-Myc (1 μg) plasmids for 36 h before infecting with EMCV at an MOI of 0.01 for 36 h. *IFN-*α and *IFN-*β mRNA levels were measured by RT-qPCR. A549 cells transfected with pCMV-Myc (1 μg) plasmid were used as control. A549 cells were transfected with pCMV-HA (0.5 μg), HA-2C (0.5 μg) or HA-3A (0.5 μg) for 24 h, and then transfected with poly (I:C) for 8 h. **(C)** *IFN-*α mRNA levels were measured by RT-qPCR. **(D)** *IFN-*β mRNA levels were measured by RT-qPCR. Data were represented as mean ± SD of three independent experiments and were measured in technical duplicate, ***P < 0.001.

Given that HSP27 can dampen EMCV infectivity, we wanted to see whether this is due to HSP27 affecting EMCV-triggered IFN response. Indeed, siRNA knockdown of HSP27 reduced EMCV-triggered *IFN-*α and *IFN-*β (Figure 5A). Consistent with this, HSP27 overexpression reversed this effect (Figure 5B). Since HSP27 can positively regulate IFN-I production triggered by EMCV, we predicted that 2C^pro^ and 3A^pro^ would also impact IFN-I production. Indeed, overexpression of 2C^pro^ and 3A^pro^ significantly blocked type I IFN production activated by poly (I:C) stimulation, synthetic dsRNA mimic (Figures 5C,D). Altogether, these findings suggest that HSP27 interacts with the type I IFN signaling cascade.

### HSP27 Enhances Encephalomyocarditis Virus-Triggered Retinoic Acid-Inducible Gene-I-Like Receptor/Melanoma Differentiation-Associated Gene 5 Signaling Pathway by Specifically Stabilizing Melanoma Differentiation-Associated Gene 5 Expression

**FIGURE 6:**
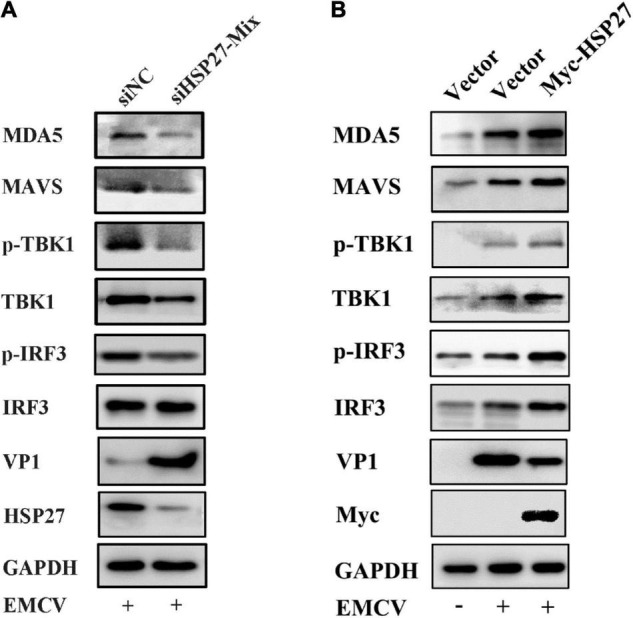
HSP27 enhances encephalomyocarditis virus (EMCV)-triggered RIG-I-like receptor (RLR)/melanoma differentiation-associated gene 5 (MDA5) signaling pathway. **(A)** A549 cells were transfected with either 150 nmol of siHSP27-Mix or siNC for 36 h before infecting with EMCV at an multiplicity of infection (MOI) of 0.01 for 36 h. The protein expression of MDA5, MAVS, TBK1, p-TBK1, IRF3, p-IRF3, HSP27, and VP1 were analyzed by immunoblotting. GAPDH was used as a loading control. **(B)** A549 cells were transfected with pCMV-Myc or Myc-HSP27 (0.5 μg) for 24 h before infecting with EMCV at an MOI of 0.01 for 36 h. The protein expression of MDA5, MAVS, TBK1, p-TBK1, IRF3, p-IRF3, Myc, and VP1 were analyzed by immunoblotting. GAPDH was used as a loading control.

**FIGURE 7:**
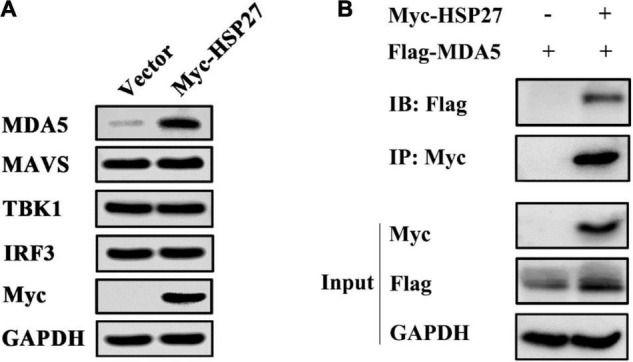
HSP27 directly interacts with melanoma differentiation-associated gene 5 (MDA5) and stabilizes MDA5 expression. **(A)** A549 cells were transfected with pCMV-Myc or Myc-HSP27 (0.75 μg) for 24 h. The protein expression of endogenous MDA5, MAVS, TBK1, IRF3, and Myc were evaluated by immunoblotting. GAPDH was used as a loading control. **(B)** A549 cells were co-transfected with Flag-MDA5 together with either pCMV-Myc or Myc-HSP27 for 36 h before performing co-immunoprecipitation (Co-IP) and immunoblotting with anti-Myc antibody. The protein expression of HSP27 and MDA5 were analyzed by immunoblotting. GAPDH was used as a loading control.

Given that EMCV mainly activates the IFN-I *via* the MDA5 signaling pathway (Gitlin et al., 2006; Kato et al., 2006), we assessed whether the positive regulatory effect of HSP27 on EMCV-triggered IFN response is due to its assistance to MDA5 signaling pathway components. Indeed, HSP27 knockdown led to a parallel loss of EMCV-triggered MDA5, MAVS, TBK1 protein expression levels (Figure 6A). Though the effect of HSP27 loss on EMCV-triggered IRF3 expression and its phosphorylation were not evident (Figure 6A), HSP27 overexpression did increase the expression of IRF3 and its phosphorylation, as well as the expression of other adaptor molecules in the MDA5 signaling pathway (Figure 6B). Consistent with the inhibitory role of HSP27 on EMCV infectivity (Figure 4), loss of HSP27 increased EMCV viral load by enhanced VP1 expression (Figure 6A). In summary, HSP27 positively regulates EMCV-triggered RLR/MDA5 signaling cascade. Given that MDA5, MAVS, TBK1, and IRF3 are activated by the same promoter, we then overexpressed HSP27 to verify the changes in the protein expression of endogenous MDA5, MAVS, TBK1, and IRF3 to reveal the specific targets of HSP27. Interestingly, we found that over-expression of HSP27 only significantly increased the protein expression of endogenous MDA5 and had no notable effect on the protein expression of endogenous MAVS, TBK1, and IRF3 (Figure 7A), indicating that HSP27 specifically stabilizes MDA5 protein expression. Co-IP assays also revealed that MDA5 directly binds to HSP27 (Figure 7B). In conclusion, we show for the first time that HSP27, as a molecular chaperone, positively regulates EMCV-triggered RLR/MDA5 signal pathway by stabilizing the expression of MDA5.

## Discussion

As a member of the *Cardiovirus* genus of the *Picornaviridae* family, EMCV is widely used as a model virus to study the mechanism of RNA virus-mediated host immune response, viral myocarditis, and insulin-dependent diabetes (Carocci and Bakkali-Kassimi, 2012). There is a growing need to elucidate the infection mechanism of EMCV for the prevention and control of EMCV. Recent studies have shown that EMCV can use its non-structural proteins L^pro^, 2B^pro^, 2C^pro^, 3C^pro^, and structural protein VP2^pro^ to inhibit type I IFN responses and degrade the key adaptor molecules in the RLRs or NF-κB pathways to escape host immune response (Li et al., 1998, 2019; Ito et al., 2012; Huang et al., 2015, 2017). Our results suggest that EMCV degrades the expression of HSP27 in host cells to evade the host immune response *via* its proteins, 2C and 3A.

Studies have shown that many members of the HSP family can affect the proliferation of a range of viruses by participating in cell growth, apoptosis, transcriptional regulation, carcinogenesis, autophagy, and innate immunity of the host (Arrigo et al., 2007; Ikwegbue et al., 2017; Zininga et al., 2018; Bolhassani and Agi, 2019; Yun et al., 2019). As one of the important members of this family of proteins, HSP27 has attracted much attention in recent years for its role in innate immune defense, viral proliferation, tumor immunity, and other immunoregulatory processes (Kumano et al., 2012; Rajaiya et al., 2012; Tong et al., 2013; Arrigo, 2017; Sun et al., 2017, 2018; Ling et al., 2018). The non-structural protein NS5A of classical swine fever virus (CSFV) can directly interact with HSP27 and thus affect the proliferation of the virus by promoting the activation of the NF-κB signaling pathway (Ling et al., 2018). It has also been suggested that HSP27 is an intracellular anti-porcine epidemic diarrhea virus factor dependent on the IFN-β signaling pathway (Sun et al., 2017). We found that overexpression of HSP27 can promote the expression of MDA5, MAVS, TBK1, and IRF3 during EMCV infection (Figure 6B), but the increased expression of MDA5, MAVS, TBK1, and IRF3 may be due to the expression of a certain plasmid in the same promoter. To verify whether HSP27 has a specific role on the expression of MDA5, MAVS, TBK1, and IRF3, we overexpressed HSP27 to detect the expression of endogenous proteins and found that HSP27 can only significantly promote the protein expression of endogenous MDA5, but did not affect the expression of endogenous MAVS, TBK1, and IRF3 (Figure 7A). Our results showed that HSP27 plays a clear antiviral role by specifically binding to MDA5 and stabilizing its expression. HSP27 exists in two forms; phosphorylated and non-phosphorylated. Non-phosphorylated HSP27 recognizes and binds to proteins damaged by oxidative stress or that have misfolded and ultimately degrades these proteins through the proteasomal pathway. On the other hand, phosphorylated HSP27 is closely related to the immune response of host cells, viral replication, proliferation, cell cycle, apoptosis, and autophagy (Arrigo, 2018). Future work aims to determine which HSP27 form is involved in EMCV pathobiology.

**FIGURE 8:**
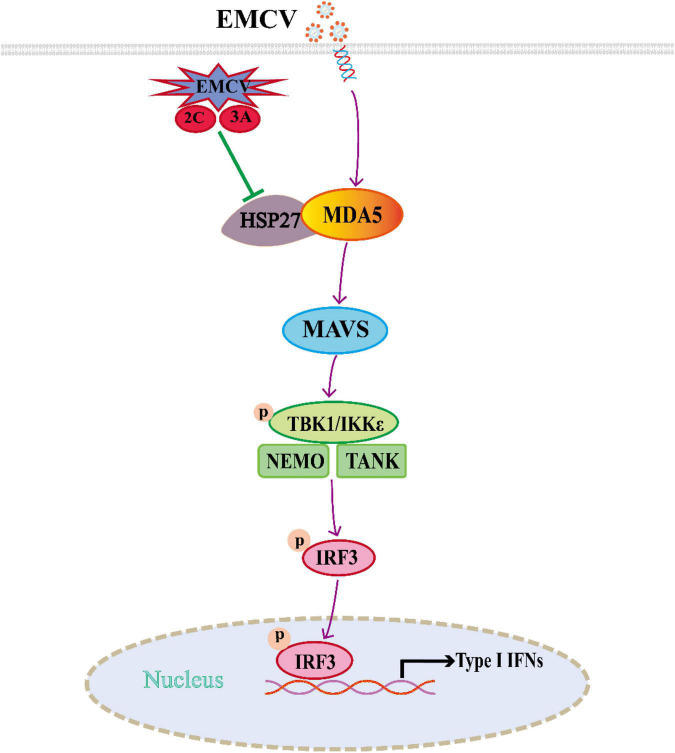
Schematic illustration of HSP27 as a molecular chaperone that stabilizes melanoma differentiation-associated gene 5 (MDA5) expression and positively regulates the type I IFN signaling pathway triggered by encephalomyocarditis virus (EMCV).

In this study, we found that EMCV 2C^pro^ and 3A^pro^ directly bind to HSP27 and reduce HSP27 protein expression, revealing a novel function of 2C^pro^ and 3A^pro^ in antagonizing the host antiviral response. We have shown for the first time that HSP27 is an important antiviral factor of EMCV infection and can be used as a potential therapeutic target against EMCV infection. In addition, it is obvious that HSP27 directly binds to and stabilizes the expression of the sensor molecule MDA5 in the RLRs signaling pathway, thereby promoting the MDA5-mediated type I IFN signaling cascade during EMCV infection (Figure 8). These findings provide new reference into the regulatory role of host factors in EMCV infection and the development of antiviral drugs and provide strong evidence for further elucidation of the molecular mechanism of EMCV pathogenesis and its immune escape strategy.

## Data Availability Statement

The original contributions presented in the study are included in the article/Supplementary Material, further inquiries can be directed to the corresponding author.

## Author Contributions

XL and RF conceived and designed the experiment. XL, RM, BW, HL, and DL performed the experiments. RM and YN analyzed the data. XL and RM wrote the original draft. AI and JX revised and proofread the draft manuscript. XL, RF, and AI finalized the manuscript. RF supervised the entire process. All authors have read and approved the final version of the manuscript.

## Conflict of Interest

The authors declare that the research was conducted in the absence of any commercial or financial relationships that could be construed as a potential conflict of interest.

## Publisher’s Note

All claims expressed in this article are solely those of the authors and do not necessarily represent those of their affiliated organizations, or those of the publisher, the editors and the reviewers. Any product that may be evaluated in this article, or claim that may be made by its manufacturer, is not guaranteed or endorsed by the publisher.

## Supplementary Material

Supplementary Figure 1 **(A–E)** A549 cells were transfected with increasing doses of HA-labeled or Myc-labeled viral proteins VP1, VP2, VP3, 2A, and 3C^pro^. Immunoblotting was used to analyze endogenous HSP27 and Myc or HA protein expression. GAPDH was used as a loading control.

Supplementary Figure 2 A549 cells were transfected with pCMV-Myc (1 μg) or Myc-HSP27 (1 μg) plasmids for 24 h before infecting with EMCV at an MOI of 0.1 for 8, 16, or 24 h. Cells and supernatant were harvested, and EMCV titers were measured by TCID_50_ assay (Reed-Muench method). Data were represented as mean ± SD of three independent experiments and were measured in technical duplicate, ^**^*P* < 0.01, ^***^*P* < 0.001.
