# Supplementary figures and images for: HSP27 Protein Dampens Encephalomyocarditis Virus Replication by Stabilizing Melanoma Differentiation-Associated Gene 5
Source: Front Microbiol. 2021 Nov 26;12:788870. doi: 10.3389/fmicb.2021.788870 (PMC8664592; doi:10.3389/fmicb.2021.788870)

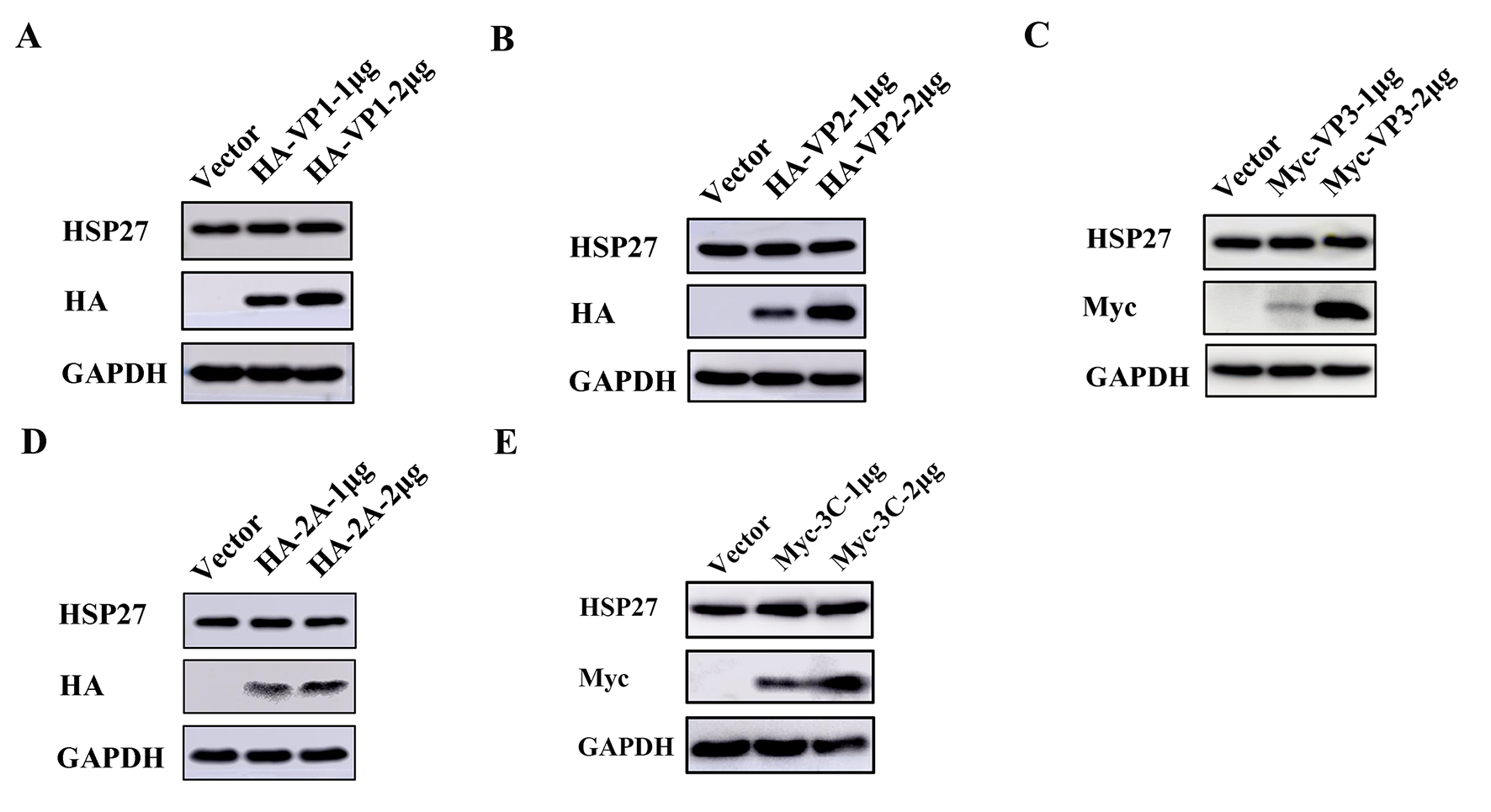

Supplement: Supplementary Figure 1 — (A–E) A549 cells were transfected with increasing doses of HA-labeled or Myc-labeled viral proteins VP1, VP2, VP3, 2A, and 3Cpro. Immunoblotting was used to analyze endogenous HSP27 and Myc or HA protein expression. GAPDH was used as a loading control. [file Image_1.TIF]

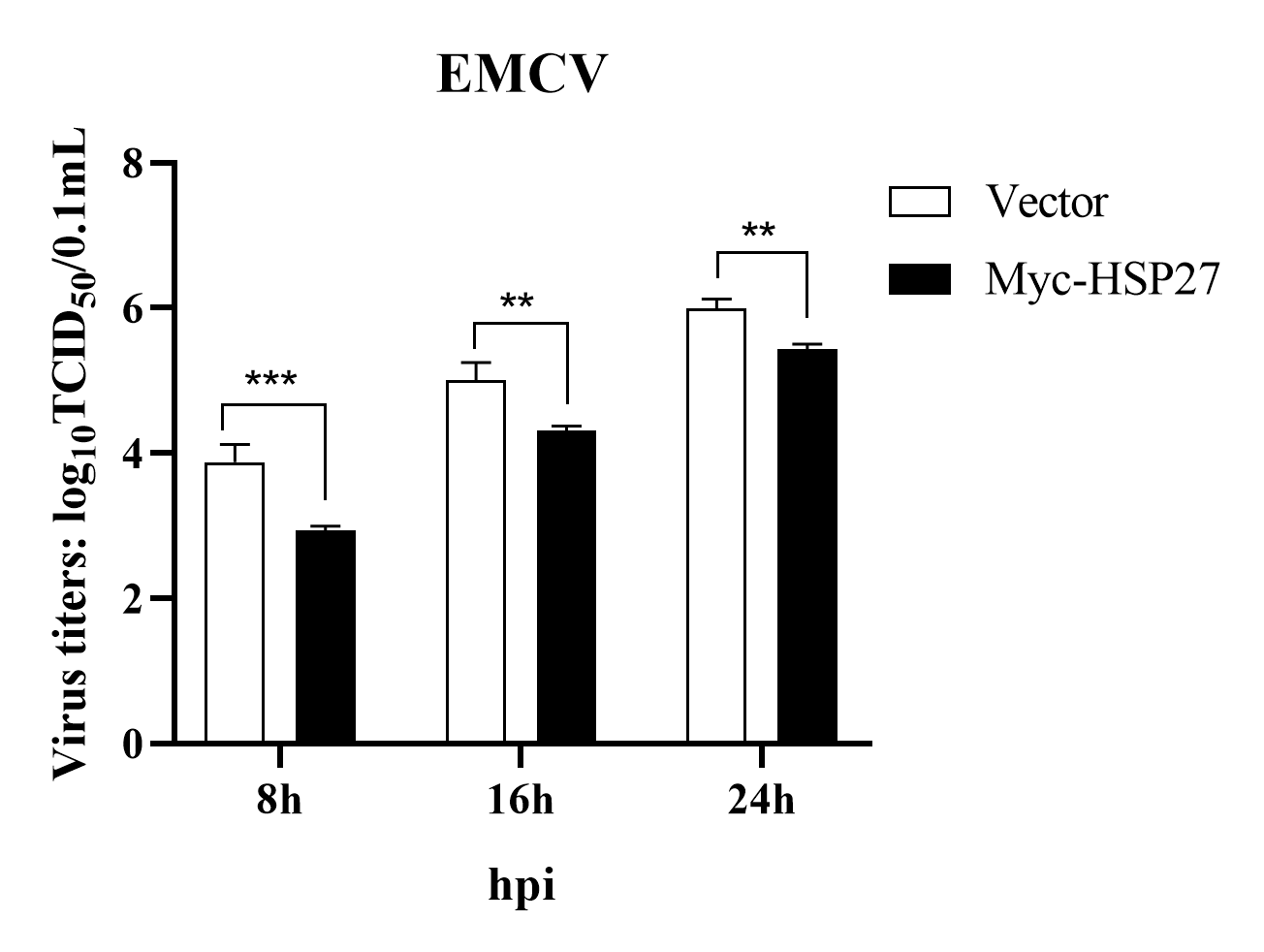

Supplement: Supplementary Figure 2 — A549 cells were transfected with pCMV-Myc (1 μg) or Myc-HSP27 (1 μg) plasmids for 24 h before infecting with EMCV at an MOI of 0.1 for 8, 16, or 24 h. Cells and supernatant were harvested, and EMCV titers were measured by TCID50 assay (Reed-Muench method). Data were represented as mean ± SD of three independent experiments and were measured in technical duplicate, **P < 0.01, ***P < 0.001. [file Image_2.TIF]
